# Associations of Sleep Quality and Awake Physical Activity with Fluctuations in Nocturnal Blood Pressure in Patients with Cardiovascular Risk Factors

**DOI:** 10.1371/journal.pone.0155116

**Published:** 2016-05-11

**Authors:** Manabu Kadoya, Hidenori Koyama, Masafumi Kurajoh, Mariko Naka, Akio Miyoshi, Akinori Kanzaki, Miki Kakutani, Takuhito Shoji, Yuji Moriwaki, Tetsuya Yamamoto, Masaaki Inaba, Mitsuyoshi Namba

**Affiliations:** 1 Department of Internal Medicine, Division of Diabetes, Endocrinology and Metabolism, Hyogo College of Medicine, 1–1 Mukogawa-cho, Nishinomiya, Hyogo 663–8501, Japan; 2 Department of Endocrinology, Metabolism and Molecular Medicine, Osaka City University Graduate School of Medicine, Osaka 545–8585, Japan; Shanghai Institute of Hypertension, CHINA

## Abstract

**Background:**

Sleep quality and awake physical activity are important behavioral factors involved in the occurrence of cardiovascular diseases, potentially through nocturnal blood pressure (BP) changes. However, the impacts of quantitatively measured sleep quality and awake physical activity on BP fluctuation, and their relationships with several candidate causal factors for nocturnal hypertension are not well elucidated.

**Methods:**

This cross-sectional study included 303 patients registered in the HSCAA study. Measurements included quantitatively determined sleep quality parameters and awake physical activity obtained by actigraph, nocturnal systolic BP (SBP) fall [100 × (1- sleep SBP/awake SBP ratio)], apnea hypopnea index, urinary sodium and cortisol secretion, plasma aldosterone concentration and renin activity, insulin resistance index, parameters of heart rate variability (HRV), and plasma brain-derived neurotrophic factor (BDNF).

**Results:**

Simple regression analysis showed that time awake after sleep onset (r = -0.150), a parameter of sleep quality, and awake physical activity (r = 0.164) were significantly correlated with nocturnal SBP fall. Among those, time awake after sleep onset (β = -0.179) and awake physical activity (β = 0.190) were significantly and independently associated with nocturnal SBP fall in multiple regression analysis. In a subgroup of patients without taking anti-hypertensive medications, both time awake after sleep onset (β = -0.336) and awake physical activity (β = 0.489) were more strongly and independently associated with nocturnal SBP falls.

**Conclusion:**

Sleep quality and awake physical activity were found to be significantly associated with nocturnal SBP fall, and that relationship was not necessarily confounded by candidate causal factors for nocturnal hypertension.

## Introduction

Behavioral and psychosocial factors have been increasingly recognized in regard to development, prevention, and treatment of cardiovascular diseases (CVD) [1–4]. Among the various behavioral factors, sleep conditions have been shown to be associated with CVD and mortality [5–8], and the potential mechanisms of these associations include impaired nocturnal blood pressure (BP) fluctuations[9]. It has been shown that deterioration in sleep quality or quantity, including short term sleep deprivation, short sleep duration, insomnia, sleep apnea, and restless legs syndrome, can contribute to occurrence of hypertension [9–11]. Furthermore, previous studies have found that ambulatory physical activity, assessed by actigraphy, is a determinant of diurnal BP variation [12–14]. However, these studies dealt with healthy subjects, work site population, or pure hypertensive subjects, and the impact of ambulatory physical activities in more general patients with cardiovascular risk factors are not examined.

In addition to sleep conditions, the underlying mechanisms of nocturnal BP variations may be diverse, including cardiac and renal function, natriuresis [15,16], the renin-angiotensin system [17,18], impaired cortisol secretion [19,20], insulin resistance [21,22], and deranged regulation of autonomic nervous activity [23–26]. Altered regulation of neuroendocrine peptides, including brain-derived neurotrophic factor (BDNF), could also contribute to diurnal BP variation [26]. However, it is not clear whether these factors underlie the mechanisms or are confounders in the association of quantitatively measures sleep quality or awake physical activity with nocturnal BP fluctuations.

To address these questions, we investigated the relationship of sleep quality or awake physical activity, based on actigraph findings, with nocturnal BP fluctuations, in conjunction with potential candidate causal factors for nocturnal hypertension including apnea-hypopnea, urinary sodium output, the renin-angiotensin-aldosterone system, urinary cortisol secretion, insulin resistance, cardiac autonomic function, and plasma BDNF in patients with cardiovascular risk factors.

## Methods

### 1. Study design and participants

All agreed to participate in the study by providing written informed consent and the study was approved by the Ethics Committee of Hyogo College of Medicine (approved No. 948). This cross-sectional study included 303 patients registered in the Hyogo Sleep Cardio-Autonomic Atherosclerosis (HSCAA) Study from October 2010 to August 2013, which was conducted to examine the impact of sleep, autonomic imbalance, and subclinical atherosclerosis on cardiovascular events [27]. Patients with one or more cardiovascular risk factor(s), (obesity, smoking, presence of cardiovascular event history, hypertension, dyslipidemia, diabetes mellitus, chronic kidney disease) and being treated at the Division of Diabetes, Endocrinology and Metabolism, Hyogo Medical College Hospital (Hyogo, Japan) were registered. None of the patients had a previous formal diagnosis of obstructive sleep apnea syndrome. Diabetic patients with insulin therapy were excluded from this study to calculate insulin resistance index, as described below.

### 2. Assessment of classical cardiovascular risk factors, cardiac and renal function, urinary samples, endocrine, and metabolic parameters

We obtained data for medical history, body mass index, and smoking status for each subject, and measured height and body weight. We defined the previous cardiovascular events as history of coronary heart disease (myocardial infarction or coronary intervention) or stroke (ischemic or hemorrhagic stroke) diagnosed by computed tomography or magnetic resonance imaging. Hypertension was defined as systolic blood pressure ≥140 mmHg, diastolic blood pressure ≥90 mmHg, or treatment for hypertension (calcium-channel blocker, angiotensin converting enzyme (ACE) inhibitor or angiotensin receptor blocker (ARB), α or β blocker and diuretic agent; Table 1). We defined dyslipidemia as the presence of low-density lipoprotein cholesterol (≥140 mg/dl), high density lipoprotein cholesterol (≤40 mg/dl), elevated triglyceride level (≥150 mg/dl), or treatment for dyslipidemia [28]. Type 2 diabetes was diagnosed based on fasting plasma glucose (FPG) ≥126 mg/dl, casual plasma glucose ≥200 mg/dl, or 2-hour plasma glucose ≥200 mg/dl during a 75-g oral glucose tolerance test, or previous therapy for diabetes [29]. Left ventricular ejection fraction (LVEF) was calculated as cardiac function from a parasternal long-axis view anteroposterior dimension measurement by cardiac ultrasonography. The estimated glomerular filtration rate (eGFR) in each patient was calculated using a new equation for Japanese subjects, as follows: eGFR (ml/min/1.73m^2^) = 194×age^-0.287^×S-Cr ^-1.094^ (if female, ×0.739)[30]. In this study, the distributions of LVEF were 56–82%, and eGFR, 48–115 ml/min. Thus, no patients with severe heart or renal failure were not included. Fasting blood samples in the morning and 24-hour urine samples were collected during measurements of ambulatory blood pressure and heart rate variability. Measurements of plasma aldosterone, plasma renin activity, and plasma BDNF were performed after resting for at least 30 minutes. Homeostasis model assessment as an index of insulin resistance (HOMA) was calculated as follows: HOMA = immunoreactive insulin (μu/ml) × FPG (mg/dL) ÷ 405. Plasma BDNF was measured as previously described [26].

**Table 1:** Clinical characteristics of subjects (n = 303).

Variables	
Age, years	58.7 ± 14.3
Male sex, n (%)	170 (56.1%)
Classical cardiovascular risk factors	
Body mass index, kg/m^2^	24.9 ± 5.2
Current smoking, n (%)	80 (26.4%)
Cardiovascular disease history, n (%)	41 (13.5%)
Hypertension, n(%)	186 (61.3%)
Calcium-channel blocker (%)	122 (40.2%)
ACE inhibitor or ARB (%)	86 (28.3%)
α or β blocker (%)	31 (10.2%)
Diuretic agent (%)	25 (8.2%)
Dyslipidemia, n (%)	187 (61.7%)
Diabetes mellitus, n (%)	117 (38.6%)
LVEF, %	67.9 ± 7.9
eGFR, ml/min/1.73m^2^	79.2 ± 22.0
ln(AHI)	1.76 ± 1.26
Urinary sodium, mEq/day	80.2 ± 32.0
Endocirne and Metabolic parameters	
Plasma aldosterone, pg/ml	107.6 ± 61.0
Plasma renin activity, ng/ml/hr	1.4 ± 1.3
Urinary cortisol, μg/day	32.7 ± 22.3
HOMA	1.9 ± 1.6
ln(BDNF), pg/ml	7.56 ± 0.85
Heart rate variability	
ln(SDNN), msec	4.76 ± 0.29
ln(CVRR), msec	2.57 ± 0.27
ln(SDANN5), msec	4.64 ± 0.32
Objective sleep/awake parameters	
ln(Sleep efficiency)	4.48 ± 0.11
ln(Sleep latency)	2.05 ± 0.67
ln(Time awake after sleep onset)	3.44 ± 0.91
ln(Wake episoded)	2.05 ± 0.67
ln(sleep physical acitivity)	2.80 ± 0.59
ln(Awake physical activity)	4.91 ± 0.33
ABPM	
SBP, mmHg	
24-Hour	123.1 ± 15.0
Sleep	116.1 ± 17.7
Awake	126.4 ± 14.9
DBP, mmHg	
24-Hour	73.6 ± 8.4
Sleep	68.5 ± 9.5
Awake	75.9 ± 8.8
Nocturnal SBP dipping, %	8.0 ± 8.4

Date are presented as mean ± standard deviation and n (%) for dichotomous variables. The parameters of HRV, BDNF, objective sleep disturbances parameters and ambulatory physical activity were natural logarithm-transformed (ln) to achieve normal distribution. ACE nenotes angiotensin converting enzyme, ARB angiotensin receprot blocker, LVEF left ventricular ejection fraction, eGFR estimated glomerular filtration rate, AHI apnea hypopnea index, HOMA homeostasis model assessment as an index of insulin resistance, BDNF brain-derived neurotrophic factor, SDNN standard deviation of the NN(RR) interval, CVRR coefficient of variation R-R interval, SDANN5 standard deviation of the average of NN intervals for each 5 min period, ABPM ambulatory blood pressure monitoring, SBP systolic blood pressure and DBP diastolic blood pressure

### 3. Determination of sleep conditions and physical activity

An actigraph (Ambulatory Monitoring, Inc., Ardley, New York, USA) was used to examine sleep quality and physical activity, as previously described [27,31]. Actigraph converts the signals produced from the acceleration sensor in samples collected at a present frequency in hertz. The samples are summed over a user-specified time sampling interval, called an “epoch”. Output from actigraph is in the form of activity “counts”. Activity “counts” are recorded by converting acceleration units over a given epoch. Subjects wore the accelerometer for 2 consecutive days. According to recommendations for clinical use of an actigraph [31], sleep physical activity (the mean of activity counts per minute by body motion during sleep), awake physical activity (the mean of activity counts per minute by walking, running and various motions during awake), sleep efficiency (percentage of time scored as sleep), sleep latency (time from lights off until time noted as sleep onset), time awake after sleep onset (number of minutes noted as awake during sleep time), and wake episodes (number of awakenings while in bed) were analyzed. Apnomonitor (SAS-2100^®^, Teijin, Tokyo, Japan) findings combined with measurements of percutaneous oxygen saturation were used to determine apnea-hypopnea index (AHI), as previously reported [26].

### 4. Cardiac autonomic function

Heart rate variability (HRV) analysis has been reported as an effective method to noninvasively measure cardiac modulation by autonomic nervous activity [32]. To examine HRV, we used an Active Tracer (AC-301A^®^, Arm Electronics, Tokyo, Japan) to monitor the surface electrocardiogram of the upper limbs for 48 hours via 3 channels, as previously described [26,27]. According to published recommendations for clinical use of HRV [32], the standard deviation of the NN(RR) interval (SDNN), coefficient of the variation R-R interval (CVRR), and standard deviation of the average NN(RR) interval for each 5-minute period (SDANN5) within the time domain were calculated.

### 5. Twenty four-hour ambulatory BP monitoring

Twenty four-hour ambulatory BP monitoring (ABPM) was performed using a TM-2431 digital recorder and obtained data were analyzed with the TM-9503 Doctor Pro 3 software package (A&D Co. Ltd., Tokyo, Japan), as previously described [26,33]. The major quality criteria used for an acceptable ABPM recording included the following: (1) minimum of 80% of the BP readings expected during the 24-hour period; (2) no more than 2 nonconsecutive hours with <1 valid BP reading; and (3) no behaviors seriously affecting BP (afternoon nap, drinking, etc.), as previously described [26]. Wake and sleep times were determined using the actigraph, as previously described [26]. Nocturnal SBP fall (%) was calculated as [100 × (1-sleep SBP/awake SBP ratio)] [12].

### 6. Statistical analysis

For analysis, sleep quality parameters, awake physical activity, and HRV parameters were natural logarithm-transformed (ln) to normalize the skewed distribution. Pearson’s correlation coefficient was used to analyze associations among factors. Multiple linear regression analyses were used to explore independent relationships among sleep quality, awake physical activity, and nocturnal SBP fluctuations. We also analyzed the impact of actigraphically-determined ambulatory physical activity in a subgroup patients without taking anti-hypertensive medications. All statistical analyses were performed using the Statistical Package for Social Sciences software package (PASW Statistics version 18.0). All reported p values are 2-tailed and were considered statistically significant at <0.05.

## Results

**Fig 1:**
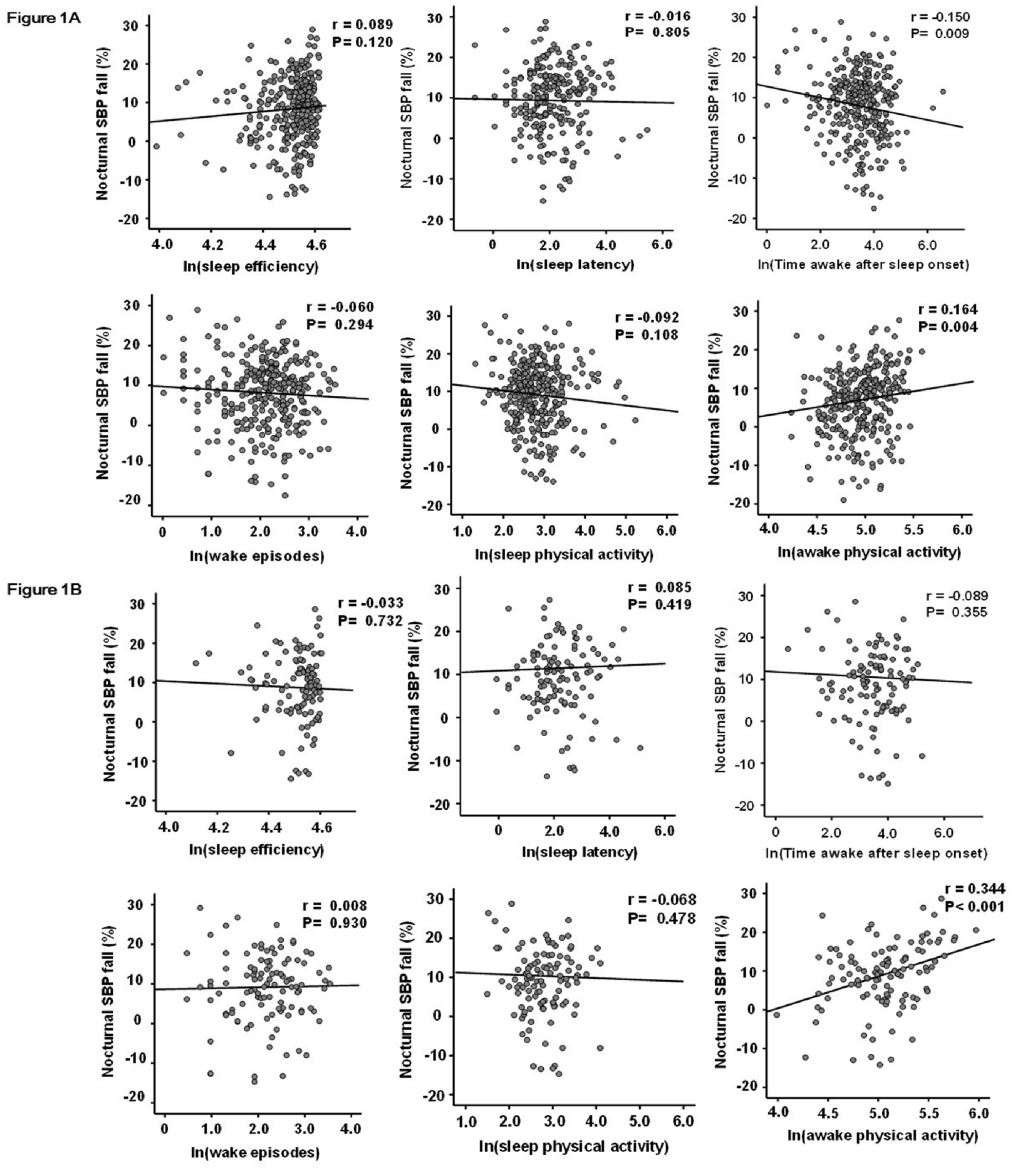
Pearson's correlation coefficients among objective parameters for sleep disturbances, ambulatory physical activity, and nocturnal SBP fall in all subjects and subgroup patients without anti-hypertensive medications. The parameters of objective sleep disturbances and ambulatory physical activity were natural logarithm-transformed (ln) to achieve a normal distribution. In all subjects, time awake after sleep onset (r = -0.150, P = 0.009) and awake physical activity (r = 0.164, P = 0.004) were significantly associated with nocturnal SBP fall (Fig 1A). In subgroup patients without anti-hypertensive medications, only awake physical activity (r = 0.344, P < 0.001) were significantly associated with nocturnal SBP fall (Fig 1B). SBP denotes systolic blood pressure. r: Pearson's correlation coefficient.

The clinical characteristics of the subjects are shown in Table 1. The correlations between sleep conditions and nocturnal SBP fall were shown in Fig 1. In all subjects, time awake after sleep onset (r = -0.150, P<0.01) and awake physical activity (r = 0.164, P<0.01) were significantly correlated with nocturnal SBP fall, while sleep efficiency, sleep latency, wake episodes, and sleep physical activity were not (Fig 1A). The associations between them were due to increased nocturnal SBP. These parameters were also significantly associated with the changes in nocturnal DBP fall (time awake sleep onset: r = -0.155, P = 0.04, awake physical activity: r = 0.209, P<0.01). In a subgroup patients without anti-hypertensive medications, awake physical activity was significantly and more strongly correlated with nocturnal SBP (r = 0.344, P<0.01) (Fig 1B) and DBP (r = 0.369, P < 0.01) falls. The associations between awake physical activity and nocturnal SBP fall in this subgroup were also due to increased nocturnal SBP. Pearson’s correlation coefficient between nocturnal SBP fall and clinical factors are shown in Table 2. The presence of ACE inhibitor or ARB treatment, eGFR, BDNF and HRV parameters (CVRR and SDANN5) were significantly correlated with nocturnal SBP fall in all subjects. Age, gender, BMI, smoking presence of past cardiovascular diseases, dyslipidemia, and diabetes were not significantly associated with nocturnal SBP fall.

**Table 2:** Correlation coefficients between nocturnal SBP fall and clinical factors.

	Medical hypertension treatment (-) (n = 117)		All (n = 303)	
Variables	r	P	r	P
Age	-0.097	0.30	-0.098	0.08
Gender (male = 1, female = 0)	-0.131	0.17	-0.036	0.52
Body mass index	0.121	0.20	-0.009	0.88
Current smoking (yes = 1. no = 0)	0.060	0.53	0.038	0.51
Cardiovascular disease history (yes = 1. no = 0)	-0.097	0.31	-0.029	0.61
Medical hypertension treatment				
Calcium-channel blocker (yes = 1. no = 0)	-	-	0.014	0.81
ACE inhibitor or ARB (yes = 1. no = 0)	-	-	-0.124	0.03
α or β blocker (yes = 1. no = 0)	-	-	-0.058	0.31
Diuretic agent (yes = 1. no = 0)	-	-	-0.070	0.22
Presence of dyslipidemia (yes = 1. no = 0)	0.129	0.17	0.066	0.25
Presence of diabetes mellitus (yes = 1. no = 0)	-0.142	0.13	-0.060	0.30
LVEF	-0.005	0.96	0.003	0.96
eGFR	0.105	0.28	0.129	0.03
ln(AHI)	-0.031	0.75	-0.112	0.06
Urinary sodium	-0.156	0.11	-0.032	0.59
Endocirne and Metabolic parameters				
Plasma aldosterone	0.069	0.48	0.048	0.42
Plasma renin activity	0.072	0.47	0.075	0.21
Urinary cortisol	0.011	0.91	0.003	0.96
HOMA	0.070	0.47	-0.021	0.92
ln(BDNF)	0.126	0.19	0.168	<0.01
Heart rate variability				
ln(SDNN)	-0.005	0.95	0.112	0.06
ln(CVRR)	0.025	0.79	0.132	0.03
ln(SDANN5)	-0.009	0.92	0.128	0.03

Pearson's correlation coefficients were performed. The parameters of AHI, BDNF and heart rate variability were natural logarithm-transformed (ln) to achieve normal distribution. ACE nenotes angiotensin converting enzyme, ARB angiotensin receprot blocker, LVEF left ventricular ejection fraction, eGFR estimated glomerular filtration rate, AHI apnea hypopnea index, SBP systolic blood pressure, HOMA homeostasis model assessment as an index of insulin resistance, BDNF brain-derived neurotrophic factor, SDNN standard deviation of the NN(RR) interval, CVRR coefficient of variation R-R interval and SDANN5 standard deviation of the average of NN intervals for each 5 min period.

Table 3 shows Pearson’s correlation coefficients of time awake after sleep onset or awake physical activity with candidate causal factors for nocturnal SBP fall (eGFR, AHI, urinary sodium, AHI, plasma aldosterone, plasma renin activity, urinary cortisol, HOMA, plasma BDNF, and HRV parameters). Of those, urinary sodium, AHI, plasma aldosterone and HRV parameters (CVRR and SDANN5) were significantly correlated with time awake after sleep onset or awake physical activity in all subjects. In patients without anti-hypertensive medications, urinary sodium and CVRR were still significantly correlated with awake physical activity.

**Table 3:** Correlation coefficients between time awake after sleep onset, awake physical activity and clinical factors.

	Medical hypertension treatment (-) (n = 117)				All (n = 303)			
	Time awake after sleep onset		Awake physical activity		Time awake after sleep onset		Awake physical activity	
Variables	r	P	r	P	r	P	r	P
LVEF	-0.043	0.66	0.099	0.32	0.017	0.77	0.088	0.14
eGFR	-0.010	0.92	0.100	0.30	-0.112	0.06	0.011	0.86
Urinary sodium	-0.093	0.34	-0.278	<0.01	-0.023	0.70	-0.221	<0.01
ln(AHI)	0.252	0.01	-0.106	0.28	0.211	<0.01	-0.072	0.22
Endocirne and Metabolic parameters								
Plasma aldosterone	-0.167	0.09	0.033	0.73	-0.126	0.03	-0.019	0.75
Plasma renin activity	0.006	0.95	-0.063	0.53	-0.071	0.23	-0.005	0.93
Urinary cortisol	0.056	0.57	0.089	0.37	0.047	0.43	-0.100	0.10
HOMA	0.142	0.15	-0.127	0.20	0.044	0.46	-0.067	0.26
ln(BDNF)	0.066	0.49	0.056	0.56	0.013	0.83	-0.077	0.19
Heart rate variability								
ln(SDNN)	-0.014	0.88	0.135	0.16	-0.110	0.07	0.128	0.03
ln(CVRR)	0.002	0.98	0.243	0.01	-0.112	0.06	0.188	<0.01
ln(SDANN5)	-0.005	0.95	0.179	0.06	-0.105	0.08	0.157	0.01

Pearson's correlation coefficients were performed. The parameters of AHI, BDNF, heart rate variability, objective sleep disturbances parameters and ambulatory physical activity were natural logarithm-transformed (ln) to achieve normal distribution. LVEF denotes left ventricular ejection fraction, eGFR estimated glomerular filtration rate, AHI apnea hypopnea index, HOMA homeostasis model assessment as an index of insulin resistance, BDNF brain-derived neurotrophic factor, SDNN standard deviation of the NN(RR) interval, CVRR coefficient of variation R-R interval and SDANN5 standard deviation of the average of NN intervals for each 5 min period.

Table 4 shows the results of multiple linear regression analysis to examine the associations of time awake after sleep onset and awake physical activity with nocturnal SBP fall, which were fully adjusted for clinical factors including potential candidate causal factors for nocturnal hypertension (age, presence of medical hypertension treatment, renal function, sleep apnea and hypopnea, urinary sodium, rennin-angiotensin system, serum cortisol, insulin resistance, plasma BDNF, cardiac autonomic function). In all subjects, those results showed that time awake after sleep onset and awake physical activity were still significantly associated with nocturnal SBP fall. Of note, in subjects without anti-hypertensive medications, time awake after sleep onset and awake physical activity were still significantly and more strongly associated with nocturnal SBP fall. Significant colinearity between these parameters was not observed (r = 0.031, P = 0.60). In addition, plasma BDNF was independently and significantly associated with nocturnal SBP fall in both subjects.

**Table 4:** Multiple linear regression analyses among sleep quality, awake physical activity and nocturnal SBP fall.

	Medical hypertension treatment (-) (n = 117)		All (n = 303)	
Variables	β	P	β	P
Age	0.162	0.26	0.174	0.05
Calcium-channel blocker (yes = 1. no = 0)	-	-	-0.036	0.60
ACE inhibitor or ARB (yes = 1. no = 0)	-	-	-0.106	0.17
α or β blocker (yes = 1. no = 0)	-	-	0.103	0.16
Diuretic agent (yes = 1. no = 0)	-	-	-0.022	0.76
eGFR	0.155	0.24	0.085	0.26
Urinary sodium	-0.091	0.43	-0.028	0.69
ln(AHI)	0.047	0.69	-0.085	0.24
Plasma aldosterone	-0.113	0.40	-0.044	0.54
Plasma renin activity	0.197	0.14	0.121	0.11
Urinary cortisol	0.045	0.69	-0.024	0.73
HOMA	0.069	0.55	0.064	0.36
ln(BDNF)	0.250	0.03	0.215	<0.01
ln(CVRR)	-0.116	0.31	0.071	0.34
ln(Time awake after sleep onset)	-0.336	0.01	-0.179	0.01
ln(Awake physical activity)	0.489	<0.01	0.190	0.01
R^2^	0.174	0.01	0.071	0.01

Multiple linear regression analyses were performed. The covariate in model included age, medical hypertension treatment, EF, eGFR, urinary sodium, AHI, plasma aldosterone, plasma renin activity, urinary cortisol, HOMA, BDNF, CVRR, wake after sleep onset and awake physical activity. AHI, BDNF, CVRR, wake after sleep onset and awake physical activity were natural logarithm-transformed (ln) to achieve normal distribution. ACE nenotes angiotensin converting enzyme, ARB angiotensin receprot blocker, eGFR estimated glomerular filtration rate, AHI apnea hypopnea index, SBP systolic blood pressure, HOMA homeostasis model assessment as an index of insulin resistance, BDNF brain-derived neurotrophic factor and CVRR coefficient of variation R-R interval. β: standard regression coefficients

## Discussion

The present study is the first to document that quantitatively measured sleep quality and awake physical activity assessed by actigraphy are associated with nocturnal SBP fall in patients with cardiovascular risk factors. Due to the cross-sectional design of the study, even though relationships among them were explored in predictive terms, the results cannot be used to show causal relationships between any of those factors. However, our study is the first to show their associations being still significant even after adjusted for other candidate causal factors for nocturnal hypertension including apnea-hypopnea, urinary sodium output, the renin-angiotensin-aldosterone system, urinary cortisol secretion, insulin resistance, cardiac autonomic function, and plasma BDNF. Moreover, the impacts of sleep quality and daytime physical activity were more prominent in a subgroup patients without taking anti-hypertensive medications.

Impaired sleep quality has been suggested to be a risk factor associated with deranged fluctuations in nocturnal BP and circadian rhythm of BP [12–14]. However, no studies have investigated through quantitatively determined sleep parameters. It is also not known whether the impact of sleep quality on nocturnal BP fluctuations is dependent or independent of other potential pathogenetic factors for nocturnal hypertension, such as natriuresis [15], the renin-angiotensin system [17,18], impaired cortisol secretion [19,20], insulin resistance [21,22], deranged autonomic nervous activity [23–26], and altered regulation of neuroendocrine peptides including brain-derived neurotrophic factor (BDNF) [26]. To directly address this issue, we quantitatively measured sleep quality, awake physical activity, and ambulatory BP monitoring together with the above-mentioned factors in 303 patients with cardiovascular risk factors who participated in the HSCAA cohort study.

Among several sleep parameters measured by actigraphy, only time awake after sleep onset was significantly associated with nocturnal SBP and DBP fall in this study. Time awake after sleep onset possibly reflects sleep fragmentation, which could be distinct from other sleep parameters, such as sleep physical activity and sleep efficiency. Increased sleep physical activity, the mean of activity counts per minute by body motion during sleep, can reflect poor sleep quality, and sleep efficiency, percentage of time scored as sleep, can estimate real sleeping time in bed. These actigraphically calculated parameters represent distinct aspects of sleep condition, and are not necessarily coordinately regulated [34]. Sleep disorders such as sleep apnea syndrome are potentially involved in the relationship between sleep quality and nocturnal BP fluctuations. Furthermore, sleep apnea was shown to be associated with decreased sleep quality [35]. Our results are in good agreement with those findings and showed that AHI was significantly correlated with parameters of sleep quality, including sleep efficiency (r = -0.170, P < 0.01), time awake after sleep onset (r = 0.211, P < 0.01), and sleep physical activity (r = 0.197, P < 0.01). Sleep apnea is also known to be associated with decreased non-rapid eye movement and increased rapid eye movement sleep, which may be primarily regulated by the autonomic nervous system [36]. Through the means of autonomic cardiovascular control, BP, heart rate, and peripheral vascular resistance progressively decrease with non-rapid eye movement sleep, with any deterioration in sleep quality or quantity possibly associated with an increase in nocturnal BP [11]. Thus, the presence of sleep apnea in conjunction with deranged autonomic regulation potentially contributes to less nocturnal BP fall in patients with poor sleep quality. In the present cohort, association of AHI with SBP fall (Table 2), and that with or DBP fall (r = -0.117, P = 0.048) was borderline significant as previously described [37]. It was also associated with decreased cardiac autonomic function when determined by measuring HRV parameters (SDNN: r = -0.217, P < 0.01; CVRR: r = -0.232, P < 0.01; SDNN5: r = -0.220, P < 0.01). The relationship between quantitatively measured sleep quality and nocturnal SBP fall was significant even after adjustment for several factors including AHI and an HRV parameter, suggesting that the impact of sleep quality on nocturnal BP fluctuations is not necessarily dependent on the presence of sleep apnea or impaired cardiac autonomic function.

Both Nocturnal SBP and DBP fall were shown as cardiovascular risk factors. SBP is mainly defined by peripheral vascular resistance, such as arterial stiffness. Autonomic dysfunction, excess sympathetic nerve functions, can increase vascular resistance, which may be associated with an increase in systolic blood pressure [38]. On the other hands, DBP is mainly defined by circulating plasma volume, such as obesity or excess sodium intakes [39]. In our cohort, DBP falls, similar to SBP falls, are significantly associated with the presence of ACE inhibitor or ARB treatment, AHI, BDNF and all HRV parameters in all subjects. Moreover, both SBP and DBP falls are independently associated with awake physical activity, or time awake after sleep onset. Unexpectedly, urinary sodium excretion or BMI is not independently associated with nocturnal DBP falls.

It has been reported that habitual physical activity is associated with lower cardiovascular mortality later in life [40]. In another study of a healthy population, physical activity, as evaluated by both accelerometer and 7-day physical activity recall questionnaire results, was found to be associated with more marked nocturnal BP dipping and, accordingly, a lower SBP and diastolic BP sleep-to-wake ratio [41]. Our findings add an important piece to those in patients with cardiovascular risk factors through quantitatively measured daytime physical activity. Actigraph has been shown to be useful to estimate the weariness in daily life [42]. The weariness induced by high physical activity in daily life may induce good sleep. In our subjects, high awake physical activity is significantly associated with good sleep efficiency (r = 0.178, P < 0.01), which may be correlated with appropriate nocturnal blood pressure fall. A previous study in healthy adults showed that habitual physical activity is associated with greater variations in beat-to-beat interval [43]. A more recent study of 985 older adults found that greater total leisure-time activity or walking alone was associated with more favorable autonomic function, including more normal circadian fluctuations and less erratic sinoatrial firing [44]. In the present study, awake physical activity was also significantly associated with autonomic function (SDNN; r = 0.128, P = 0.03, CVRR; r = 0.188, P < 0.01, SDANN5; r = 0.157, P < 0.01). Of importance, the relationship between awake physical activity and nocturnal BP fall was still significant after adjustment for other potential confounders including HRV parameters. Moreover, even though daytime physical activity and sleep quality are mutually interrelated, both parameters were independently correlated with nocturnal BP fall.

We examined several clinical parameters shown to be involved in nocturnal hypertension, including LVEF, eGFR, insulin resistance index HOMA, plasma aldosterone, plasma rennin activity, plasma BDNF, urinary cortisol, and urinary sodium output. Among those, HOMA, plasma aldosterone, plasma rennin activity, urinary cortisol, and urinary sodium were not significantly associated with nocturnal BP fall in either simple or multiple regression analysis results in all or subgroup subjects without taking anti-hypertension medications (Table 2). eGFR was significantly and positively associated with nocturnal BP fall in simple regression analysis, though significance was lost after adjustment for other clinical factors. In addition, plasma BDNF was one of the factors significantly and positively correlated with nocturnal SBP fall in simple regression analysis, as previously reported [26]. Moreover, the correlation remained significant even after other clinical parameters including sleep and physical activity were included as covariates in multiple regression analyses. On the other hand, the association of sleep quality or awake physical activity with nocturnal BP fall was significant even after adjustment for plasma BDNF level. Taken together with the very low R^2^ values (as high as 0.071) in multiple regression analysis for determining nocturnal BP fall, the candidate clinical factors identified thus far for nocturnal hypertension including plasma BDNF do not explain the underlying mechanisms of the association between sleep quality or awake physical activity and nocturnal BP fall. Of note however, in subgroup patients without anti-hypertensive medications, much higher values for sleep quality and daytime physical activity for independent associations with nocturnal SBP falls, with larger R^2^ value of 0.174, suggesting that anti-hypertensive medications could underscore the impact of these parameters on BP fluctuation. Anyhow, unidentified mechanisms apparently remain in the pathogenesis of nocturnal BP fluctuation.

Our study has several limitations. First and most importantly, due to the cohort design, we could not measure circadian changes in plasma or urinary hormones and electrolytes, such as circadian cortisol rhythm or daytime-nighttime natriuresis, which may be more directly associated with circadian BP fluctuations [16,20]. Second, even though the number of subjects was adequate to examine associations among factors, our cohort size cannot be generalized to all patients with cardiovascular risk factors. Third, because of ethical considerations, our subjects were analyzed for patterns of nocturnal blood pressure fluctuations without discontinuation of antihypertensive medication, which is generally performed for ABPM measurements in a healthy population. Although we performed analysis in a subgroup of patients without anti-hypertensive medications and obtained impressive results, the numbers of subjects in the group may not be sufficient to get conclusive data. Finally, diabetic neuropathy was not included as a covariate in this study, because quantitative nerve conduction velocity was not measured in all of the diabetic subjects. Nevertheless, our findings regarding mutual relationships among sleep quality, awake physical activity, and nocturnal SBP fall are important to answer pressing pathophysiological questions in regard to hypertension. These correlations could potentially be existed if life stress on some subjects induced both higher nocturnal blood pressure and lower sleep quality. With the findings of the strong impact of behavior factors on BP fluctuation independent of classical predictors of nocturnal hypertension such as renin-aldosterone system, insulin resistance, natriuresis, and apnea or hypopnea, we may need to emphasize more the usefulness of behavioral education including daily exercise and good sleep conditions for the treatment and prevention of nocturnal hypertension in a regular clinical setting.
